# Analysis of two layered peristaltic-ciliary transport of Jeffrey fluid and in vitro preimplantation embryo development

**DOI:** 10.1038/s41598-024-51641-3

**Published:** 2024-01-17

**Authors:** Hameed Ashraf, Imran Siddique, Ayesha Siddiqa, Ferdous M. O. Tawfiq, Fairouz Tchier, Rana Muhammad Zulqarnain, Hamood Ur Rehman, Shahzad Bhatti, Abida Rehman

**Affiliations:** 1 Department of Mathematics, University of Okara, Okara, Pakistan; 2 Department of Mathematics, University of Sargodha, Sargodha, 40100 Pakistan; 3 Department of Mathematics, University of Management and Technology, Lahore, 54770 Pakistan; 4 Mathematics Department, College of Science, King Saud University, P.O. Box 22452, Riyadh, 11495 Saudi Arabia; 5 School of Mathematical Sciences, Zhejiang Normal University, Jinhua, 321004 Zhenjiang China; 6 Lahore Institute of Fertility & Endocrinology, Hameed Latif Hospital, Lahore, Pakistan; 7 Postgraduate Resident MD Pediatrics, The University of Child Health and Sciences (Children Hospital), Lahore, Pakistan

**Keywords:** Ecology, Mathematics and computing

## Abstract

The analysis of peristaltic-ciliary transport in the human female fallopian tube, specifically in relation to the growing embryo, is a matter of considerable physiological importance. This paper proposes a biomechanical model that incorporates a finite permeable tube consisting of two layers, where the Jeffrey fluid model characterizes the viscoelastic properties of the growing embryo and continuously secreting fluid. Jeffrey fluid entering with some negative pressure gradient forms the core fluid layer while continuously secreting Jeffrey fluid forms the peripheral fluid layer. The resulting partial differential equations are solved for closed-form solutions after employing the assumption of long wavelength. The analysis delineated that increasing the constant secretion velocity, Darcy number, and Reynolds number leads to a decrease in the appropriate residue time of the core fluid layer and a reduction in the size of the secreting fluid bolus in the peripheral fluid layer. Eventually, the boluses completely disappear when the constant secretion velocity exceeds 3.0 Progesterone ($P_4$) and estradiol ($E_2$) directly regulate the transportation of the growing embryo, while luteinizing hormone (LH) and follicle-stimulating hormone (FSH), prolactin, anti-mullerian hormone (AMH), and thyroid-stimulating hormone (TSH) have an indirect effects. Based on the number and size of blastomeres, the percentage of fragmentation, and the presence of multinucleated blastomeres two groups were formed in an in vitro experiment. Out of 50 patients, 26 (76.5%) were pregnant in a group of the good quality embryos, and only 8 (23.5%) were in a group of the bad quality embryos. The transport of growing embryo in the human fallopian tube and preimplantation development of human embryos in in vitro are constraint by baseline hormones FSH, LH, prolactin, $E_{2}$, AMH, and TSH.

## Introduction

The cervix, uterus, fallopian tube, and ovaries form a functional complex devoted to the reproduction of human beings. The primary means of transportation in the cervix, uterus, and ovary is because of the peristaltic contractions of smooth muscle cells. On the other hand, in the fallopian tube, the swaying motions of the ciliary cells and the cyclic peristaltic contractions of the smooth muscle cells are sources of transportation. The peristaltic contractions and swaying motions give rise to a sinusoidal wave and a metachronal wave, respectively. These two motions are interconnected and together produce a travelling wave due to their continuous nature. In turn, this travelling wave is responsible for transportation in the fallopian tube. The cervix, uterus, and fallopian tube provide passage to the self-propelling spermatozoa in the preovulatory phase. Ovaries produce ovum and ovulate them at the time of ovulation. The vital role of the fallopian tubes now comes into play. The fallopian tube consists of four regions: the infundibulum, ampulla, isthmus, and intramural. Funnel-shaped fimbriae at the distal end of the infundibulum spread out over the ovary and capture the ovum from the surface of the ovary. The ovum from the infundibulum region to the entrance of the ampullar region enters with some negative pressure gradient. Fertilization mostly takes place in this region. When fertilization occurs in this area, the travelling wave aids in transporting the growing embryo from the ampullary region to the isthmus and eventually to the intramural region. Finally, the growing embryo leaves the intramural and enters into the uterus, where, with the further assistance of peristalsis of the uterus, it is to be transported to the site of implantation for a successful pregnancy. Subsequently, one cannot neglect the crucial role of a functional human fallopian tube in the successful reproduction of a human being, and thus it cannot be overlooked. Thus, understanding the basic developmental aspects of human preimplantation provides biological insight into human development and some common birth defects. It also comprehends the potential benefits for reproductive health and improvements in reproductive medicine^1–7^.

Most studies of human preimplantation available in the literature have been carried out in the context of in vitro fertilization (IVF)^8–13^. With the help of sensitive gene expression profiling technologies and advanced imaging techniques, in vitro successfully provided some insight into the developmental aspects of human pre-implantation. In vitro is a type of assisted reproductive technology which involves ovum retrieval from human female ovaries and spermatozoa collection from a human male. When spermatozoa and ovum are placed together in a petri dish, then the process of insemination takes place. This petri dish is then kept in an environmentally controlled chamber. More often, after a few hours of insemination, a spermatozoon fertilizes an ovum. In some cases, if the technician thinks that the chance of fertilization is low, then he may inject a spermatozoon directly into the ovum. This process in literature is called “intracytoplasmic spermatozoon injection (ICSI)”. To ensure that the growing embryo is undergoing mitotic divisions properly technician monitors the petri dish and may perform a few tests for genetic conditions. After 3 to 5 days of culture, the growing embryo is then to be transferred using a catheter into the uterus for implantation to develop pregnancy^14,15^.

When a female reaches puberty, in the hypothalamus of the brain, neurosecretory cells synthesize a gonadotropin-releasing hormone (GRH). The GRH in turn stimulates the release of luteinizing hormone (LH) and follicle-stimulating hormone (FSH) in the pituitary gland. At the time of ovulation, as the fimbriae capture an ovum within the ovary corpus luteum, a temporary gland secretes progesterone and estrogen. LH has more prominent effects on ovulation and progesterone production. On the other hand, FSH contributes to the maturation of follicles and the production of estrogen. If a spermatozoon fertilizes an ovum, then the secretion of progesterone and estrogen increases. These hormones prevent the ovum from maturing further^16^. Estradiol ($E_{2}$) and progesterone ($P_{4}$) are the most abundant and major types of estrogen and progestogen respectively. Lactotrop cells of the pituitary gland, mammary gland, uterus, lymphocytes, and placenta synthesize and secrete prolactin. It induces and regulate the estradiol ($E_{2}$) and progesterone ($P_{4}$) synthesis. In turn, $E_{2}$ and $P_{4}$ through specific hormone receptors play an important role in the normal functioning of the smooth muscle cells, ciliary cells, and goblet cells^17,18^. Anti-mullerian hormone (AMH) is a dimeric glycoprotein belonging to the transforming growth factor-$\beta$ family. AMH serves as an indicator of the quantity and quality of ovarian follicles. An ovarian reserve may be diminished when AMH levels are low, potentially affecting follicular development and ovulation. If follicular development slows down, it could affect how the ovum is released and transported into the fallopian tubes. Thus, AMH levels can indirectly regulate the transport of a growing embryo^19–21^. The thyroid gland secretes thyroid-stimulating hormone (TSH). TSH is a heterodimeric glycoprotein hormone that shares the $\alpha$-subunit with GRH, FSH, and LH. The secretion of TSH is also under the influence of $E_{2}$ and prolactin. TSH employs its effects through the TSH receptor found in the cell membrane of thyroid follicular cells. It plays an essential role in preimplantation embryonic development in the human female fallopian tube^22,23^.

The goblet cells lining the epithelium of the fallopian tube are the unicellular cells. These cells are specialized for the continuous secretion of viscoelastic fluid. The secretion of a viscoelastic fluid is a continuous process, which comprises a combination of secretory products and their corresponding polyionic charges. Secretory products include glucose, immunoglobulins, albumin, amino acid, pyruvate, and lactate and their polyionic charges include Zn$^{2+}$, Mg$^{2+}$, Na$^{+}$, K$^{+}$, Ca$^{2+}$, S$^{2-}$, Cl$^{-}$, and HCO$_{3}^{-}$. It also includes some growth factors: fibroblast growth factors, epidermal growth factors, transforming growth factors, and insulin-like growth factors^24–27^. In biomechanics, we use the concept of permeable surfaces that are accompanied by injection. This concept is equivalent to the concept of continuous secretion of viscoelastic fluid from the goblet cells of the fallopian tube^28–30^. When fluid comes in contact with permeable surface then the fluid and the boundary move with different velocity. This situation in biomechanics gives rise to the slip boundary condition. Beaver and Joseph^31^ experimentally analysed the flow at the interface between a porous medium and fluid layer. Saffman^32^ improved the boundary condition of Beaver and Joseph^31^. He proposed a boundary condition that also provides the theoretical justification of the Beaver and Joseph proposed slip boundary condition. This slip boundary condition in literature is known as Saffman boundary condition^33,34^.

Nowadays, it has been well established that most biofluids behave like non-Newtonian viscoelastic fluids^35^. Various researchers were interested in the Jeffrey fluid model because it was thought to be better for biofluids than other non-Newtonian viscoelastic fluid models. Jeffrey’s model is the simplest non-Newtonian model, which uses time derivatives rather than convective derivatives and exhibits time delay with relaxation behaviour. It has the ability to capture the viscoelastic properties of some physiological phenomena in the human body. The authors are now concentrating on this model, as it is the simplest non-Newtonian viscoelastic fluid model. The things they looked at were the movement of Jeffrey fluid, which is urine from the kidney to the bladder, chyme in the digestive tract, a few worms, lymph in the lymphatic vessels, spermatozoa in the male reproductive tract’s ducts, and ovum in the female fallopian tube^36–50^. Therefore, to analyze the growing human embryo transport in the human fallopian tube, the Jeffrey fluid model would be the better choice.

In their study, Ashraf et al.^1^ proposed a biomechanical model that illustrated how a pressure gradient and a propagating travelling wave can generate a linearly viscous biofluid flow within a uniform, finite, narrow tube. They termed such biofluid flow as “peristaltic-ciliary flow”. Later, they characterized the viscoelastic properties of the secreted fluid and growing embryos using different fluid models, including the Powell–Eyring, Johnson–Segalman, Carreau, and third grade fluid models^2–5^. They relate the findings of their study with the transport of growing embryos within the female human fallopian tube to provide their significance. In their model, they considered that goblet cells poured out a small quantity of the viscoelastic fluid in spite of the fact that goblet cells secrete viscoelastic fluid continuously. To the author’s knowledge previously no attempt has been made to analyze the peristaltic-ciliary transport of incompressible Jeffrey fluids in two immiscible fluid layers: peripheral fluid layer and core fluid layer in a finite permeable narrow tube. The permeable surface of the tube continuously secretes (injects) Jeffrey fluid in the peripheral fluid layer.

This paper aims to delve further into the transport of human embryos within the fallopian tube by improving the biomechanical model of Ashraf et al.^1–5^. We make improvements in this biomechanical model by replacing the nonuniform, finite, narrow tube with a uniform permeable, finite, narrow tube. The permeable surface of the tube continuously secretes Jeffrey fluid with constant velocity. The peristaltic-ciliary flow of two immiscible Jeffrey fluids of different viscosities and densities in this permeable tube is induced by the pressure gradient at the permeable tube entrance and the propagating peripheral and core travelling waves. We will make use of the assumption of long wavelength to simplify the formulated system of partial differential equations. The closed-form solutions of the simplified system of partial differential equations will be obtained. We will derive exact analytical expressions for flow variables such as pressure gradient, stream functions, and axial and radial velocity components. Through numerical integration in MATHEMATICA, appropriate residue will be computed. In this study, we will look at how different fluid model parameters, geometric parameters, condition of pressure gradient at the permeable entrance tube, and the Reynolds number affect the appropriate residue time of the core fluid layer and the size of the trapped boluses in the peripheral fluid layer. We will also discuss the human embryo preimplantation developments in the in vitro context by taking phase-contrast images of cultured embryos in the lab. Furthermore, we will explore the effects of progesterone $P_4$, $E_2$, LH, FSH, prolactin, AMH, and TSH on both the peripheral travelling wave and core travelling wave dynamics. Additionally, we will delve into the effects of these factors on the development of human embryos during the preimplantation stage through in vitro experimental data.

## Problem formulation and mathematical modelling

**Figure 1:**
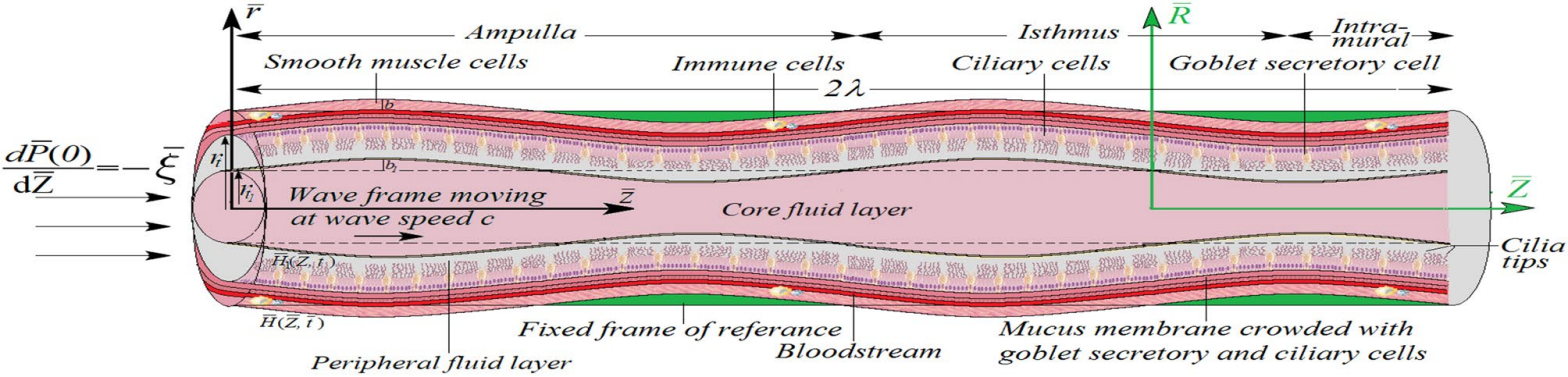
Schematic diagram of the problem.

We consider the peristaltic-ciliary transport of incompressible Jeffrey fluids with different viscosities and densities in two immiscible fluid layers in a finite permeable narrow tube. It is assumed hypothetically that a uniform cross-section permeable tube consists of the ampullar, isthmus, and intramural regions whose surface is lined with a mucus membrane having serosa, myosalpinx, and endosalpinx as three different layers. The outermost layer of the mucus membrane lining the permeable tube is known as the serosa. The intermediate layer called the myosalpinx, is made up of groups of smooth muscle cells. On the apex of the endosalpinx, two types of uniformly distributed epithelial cells: ciliary cells and goblet cells are situated. The goblet cells continuously secrete viscoelastic fluid with constant secreting velocity. Here we use the concept that the permeable surface of the tube is accompanied by the continuous injection of fluid that forms a peripheral region called the “peripheral fluid layer”. Fluid entering with some negative pressure gradient, $-{\overline{\xi }}$ forms a core region called the “core fluid layer”. We use Jeffrey fluid model to characterize the peripheral fluid layer and core fluid layer. A schematic diagram is shown in Fig. 1 to illustrate the tube’s structure and dimensions. The length of this tube is ${\bar{L}}=2 \lambda$. This diagram shows a coordinate system that uses cylindrical coordinates to represent the system within the laboratory reference frame $\left( {\bar{R}}, {\bar{Z}}, {\bar{t}}\right)$ in which the origin is taken at the midplane, the ${\bar{Z}}$-axis along the tube while ${\bar{R}}$-axis normal to the tube. The tube consists of two regions: peripheral region of mean radius $r_{t}$ ($-r_{t}\le {\bar{R}} \le r_{t}$) and core region of mean radius $r_{t_1}$ ($-r_{t_1}\le {\bar{R}} \le r_{t_1}$).

The smooth muscle cells’ peristaltic contractions and ciliary cells’ swaying motions generate sinusoidal and metachronal waves, respectively, in the system. These waves in turn give rise to propagating travelling wave called the peripheral travelling wave ${\bar{H}}\left( {\bar{Z}},{\bar{t}}\right)$:

1 $$\begin{aligned} {\bar{H}}\left( {\bar{Z}},{\bar{t}}\right) ={r_{t}}+b\sin \frac{2\pi }{\lambda }\left( {\bar{Z}}-{\text{ v }}{\bar{t}}\right) +Ab\frac{2\pi }{\lambda }\cos \frac{2\pi }{\lambda }\left( \kappa \left( {\bar{Z}}-{\text{ v }}{\bar{t}}\right) \right) \sin \frac{2\pi }{\lambda }\left( {\bar{Z}}-{\text{ v }}{\bar{t}} \right) , \end{aligned}$$

here we denote ${\bar{t}}$ for any instant of time, $\text{ v }$ for the wave speed, $\kappa$ for the constant, *b* for the amplitude, $\lambda$ for the wavelength, and *Ab* for the maximum displacement of the material points of ciliary cells. The interface between the peripheral fluid layer and the core fluid layer exhibits similar characteristics to the peripheral travelling wave ${\bar{H}}\left( {\bar{Z}},{\bar{t}}\right)$ (1) due to their shared adherence to the same fundamental principles of physics. The equilibrium of the forces acting on the fluid determines the wave’s configuration. The geometry of the interface between the two fluids is determined by the same balance of forces that defines the shape of the peripheral travelling wave^51^. Hence, it is logical for us to consider the propagating travelling wave at the interface as the core travelling wave ${\bar{H}}_{1}\left( {\bar{Z}},{\bar{t}}\right)$:

2 $$\begin{aligned} \bar{H_{1}}\left( {\bar{Z}},{\bar{t}}\right) =r_{t_{1}}+b_{1}\sin \frac{2\pi }{ \lambda }\left( {\bar{Z}}-\text{ v }{\bar{t}}\right) +A_{1}b_{1}\frac{2\pi }{\lambda }\cos \frac{2\pi }{\lambda }\left( \kappa \left( {\bar{Z}}-\text{ v }{\bar{t}}\right) \right) \sin \frac{2\pi }{\lambda }\left( {\bar{Z}}-\text{ v }{\bar{t}} \right) . \end{aligned}$$

For the core travelling wave ${\bar{H}}_{1}\left( {\bar{Z}},{\bar{t}}\right)$, we use the $b_{1}$ and $A_{1}b_{1}$ to denote the amplitude and maximum displacement of the material points of ciliary cells, respectively^1–5^. Along the permeable tube surface at a wave-speed $\text{ v }$, both these travelling waves propagate with different amplitudes in the positive ${\bar{Z}}$-axis direction and drive the two-dimensional flow of two immiscible Jeffrey layersperipheral fluid layer and core fluid layerin the permeable tube. Velocity vector that can describe the motion of such immiscible fluid flow is of the form:

3 $$\begin{aligned} {\varvec{{\bar{V}}}}_{k}=\left[ {\bar{U}}_{k}\left( {\bar{R}},{\bar{Z}},{\bar{t}}\right) ,0,{\bar{W}}_{k}\left( {\bar{R}}, {\bar{Z}},{\bar{t}}\right) \right] , \qquad \text{ where } \qquad k=p,c, \end{aligned}$$

where we denote $({\bar{U}}_{k},{\bar{W}}_{k})$ for (axial, radial) components of velocity while $`p{\text{'}}$ for peripheral region and $`c{\text{'}}$ for core region. We neglect the thermal effects and body forces. The flow under consideration can be governed by the continuity equation and momentum balance along with the extra stress tensor for incompressible Jeffrey fluid in the laboratory reference frame^52–55^:

4 $$\begin{aligned} \dfrac{\partial }{\partial {\bar{R}}}\left( {\bar{R}}{\bar{U}}_{k}\right)&=-{\bar{R}}\dfrac{\partial {\bar{W}}_{k}}{\partial {\bar{Z}}}, \end{aligned}$$

5 $$\begin{aligned} \rho _{k} \dfrac{{\bar{D}}\bar{U_{k}}}{{\bar{D}}{\bar{t}}}&= -\dfrac{\partial {\bar{P}}}{\partial {\bar{R}}}+\dfrac{1}{{\bar{R}}}\dfrac{\partial }{\partial {\bar{R}}}\left( {\bar{R}}{\bar{S}}_{{\bar{R}}{\bar{R}}}^{k}\right) + \dfrac{\partial }{\partial {\bar{Z}}} \left( {\bar{S}}_{{\bar{R}}{\bar{Z}}}^{k}\right) - \dfrac{{\bar{S}}_{{\bar{\theta }}{\bar{\theta }}}^{k}}{{\bar{R}}}, \end{aligned}$$

6 $$\begin{aligned} \rho _{k} \dfrac{{\bar{D}}{\bar{W}}_{k}}{{\bar{D}}{\bar{t}}}&= -\dfrac{\partial {\bar{P}}}{\partial {\bar{Z}}}+\dfrac{1}{{\bar{R}}}\dfrac{\partial }{\partial {\bar{R}}} \left( {\bar{R}}{\bar{S}}_{{\bar{Z}}{\bar{R}}}^{k}\right) +\dfrac{\partial }{\partial {\bar{Z}}}\left( {\bar{S}}_{{\bar{Z}}{\bar{Z}}}^{k}\right) , \end{aligned}$$

7 $$\begin{aligned} {\varvec{{\bar{S}}}^{k}}=\frac{\mu _{k}}{1+\lambda _{1}^{k}} \left[ {\bar{\mathbf {A}}}_{1}^{k}+\lambda _{2}^{k}\dfrac{ D{\bar{\mathbf {A}}}_{1}^{k}}{D{\bar{t}}}\right] , \end{aligned}$$

where we denote ${\bar{\theta }}$ for azimuthal coordinate, $\frac{{\bar{D}}}{{\bar{D}}{\bar{t}}}$ for material time derivative, $\rho _{k}$ for constant density of the fluid, ${\bar{P}}$ for pressure, ${\bar{S}}^{k}_{{\bar{R}}{\bar{R}}}$, ${\bar{S}}^{k}_{{\bar{R}}{\bar{Z}}}$, ${\bar{S}}^{k}_{{\bar{\theta }} {\bar{\theta }}}$, ${\bar{S}}^{k}_{{\bar{Z}}{\bar{R}}}$ and ${\bar{S}}^{k}_{{\bar{Z}}{\bar{Z}}}$ for components of extra stress tensor ${\varvec{{\bar{S}}}^{k}}$, ${\bar{\mathbf {A}}}_{1}^{k}$ for first Rivlin–Ericksen tensor, $\lambda _{1}^{k}$ for ratio of relaxation time to retardation time, $\lambda _{2}^{k}$ for retardation time and $\mu _{k}$ for dynamic viscosity.

First Rivlin–Ericksen tensor ${\varvec{{\bar{A}}}}_{1}^{k}$ is defined as

8 $$\begin{aligned} {\varvec{{\bar{A}}}}_{1}^{k}=grad{\bar{\mathbf{V}}}_{k}+\left( grad{\bar{\mathbf{V}}}_{k}\right) ^{T}, \end{aligned}$$

in which *T* denotes for transpose and

$$\begin{aligned} grad{\bar{\mathbf{V}}}_{k}=\left[ \begin{array}{ccc} \dfrac{\partial {\bar{U}}_{k}}{\partial {\bar{R}}}&{} 0&{} \dfrac{\partial {\bar{U}}_{k}}{\partial {\bar{Z}}} \\ 0 &{}{\bar{R}}^{-1}{\bar{U}}_{k} &{} 0 \\ \dfrac{\partial {\bar{W}}_{k}}{\partial {\bar{R}}} &{} 0 &{} \dfrac{\partial {\bar{U}}_{k}}{\partial {\bar{Z}}} \\ \end{array} \right] . \end{aligned}$$.

*Remark*: An extra stress tensor can be obtained for a fluid with linear viscosity on setting $\lambda _{1}^{k}=\lambda _{2}^{k}=0$ in Eq. (7).

With some negative pressure gradient, $-{\bar{\xi }}$ the two immiscible Jeffrey peripheral fluid layer and core fluid layer enter at the entrance of the permeable tube and fills it completely^1–5^. Therefore, we can use the condition of pressure gradient at the permeable tube entrance:

9 $$\begin{aligned} \dfrac{\partial {\bar{P}}({\bar{Z}}=0,{\bar{t}})}{\partial {\bar{Z}}}=-{\bar{\xi }}. \end{aligned}$$

The surface of the permeable tube is denoted by ${\bar{R}}={\bar{H}}\left( {\bar{Z}}, {\bar{t}}\right)$ in the schematic diagram (refer to Fig. 1) and assume that it moves solely perpendicular to the ${\bar{Z}}$-axis only. The two immiscible Jeffrey peripheral fluid layer and core fluid layer in a permeable narrow tube can be described by the following boundary conditions: At ${\bar{R}}=0$

10 $$\begin{aligned} {\bar{S}}^{c}_{{\bar{R}}{\bar{Z}}}\left( 0, {\bar{Z}}, {\bar{t}}\right)&=0, \end{aligned}$$

11 $$\begin{aligned} {\bar{U}}_{c} \left( 0, {\bar{Z}}, {\bar{t}}\right)&=0. \end{aligned}$$

At $${\bar{R}}=H_{1}$$

12 $$\begin{aligned} {\bar{W}}_{p}\left( {\bar{H}}_{1}, {\bar{Z}}, {\bar{t}}\right)&={\bar{W}}_{c}\left( {\bar{H}}_{1}, {\bar{Z}}, {\bar{t}}\right) , \end{aligned}$$

13 $$\begin{aligned} {\bar{U}}_{p}\left( {\bar{H}}_{1}, {\bar{Z}}, {\bar{t}}\right)&={\bar{U}}_{c}\left( {\bar{H}}_{1}, {\bar{Z}}, {\bar{t}}\right) , \end{aligned}$$

14 $$\begin{aligned} {\bar{S}}^{p}_{{\bar{R}}{\bar{Z}}}\left( {\bar{H}}_{1}, {\bar{Z}}, {\bar{t}}\right)&={\bar{S}}^{c}_{{\bar{R}}{\bar{Z}}}\left( {\bar{H}}_{1}, {\bar{Z}}, {\bar{t}}\right) . \end{aligned}$$

At $${\bar{R}}=H$$

15 $$\begin{aligned} {\bar{W}}_{p}\left( {\bar{H}}, {\bar{Z}}, {\bar{t}}\right)&=-\dfrac{\sqrt{K}}{\beta }\dfrac{\partial }{\partial {\bar{R}}} {\bar{W}}_{p}\left( {\bar{H}}, {\bar{Z}}, {\bar{t}}\right) , \end{aligned}$$

16 $$\begin{aligned} {\bar{U}}_{p}\left( {\bar{H}}, {\bar{Z}}, {\bar{t}}\right)&=\dfrac{\partial {\bar{R}}}{\partial {\bar{t}}}, \end{aligned}$$

where we represent *K* for permeability of the porous medium and $\beta$ for slip parameter.

We use the symbols $r_{t}$ to denote the characteristic length scale in the depthwise direction, $\lambda$ to denote the characteristic length scale in the streamwise direction, $\text{ v }$ to denote the characteristic velocity scale, and $\lambda /\text{v }$ to denote the characteristic time scale. Now, we adopt a moving reference frame $\left( {\bar{r}}, {\bar{z}}\right)$ that moves at a speed of $\text{ v }$ in the positive axial direction with respect to the laboratory reference frame. In this reference frame, the flow of two immiscible layersthe peripheral fluid layer and the core fluid layerin a permeable, narrow tube becomes time-independent. We use a dimensionless approach to scale the transformations between these two frames in following way:

17 $$\begin{aligned} \begin{aligned} r&=\dfrac{{\bar{R}}}{r_{t}}, \quad z=\dfrac{2\pi \left( {{\bar{Z}}}-\text{ v }{\bar{t}}\right) }{\lambda }, \quad w_{k}=\dfrac{\bar{W_{k}}-\text{ v }}{\text{ v }}, \quad u_{k}=\dfrac{\bar{U_{k}}\lambda }{2\pi \text{ v } r_{t}}, \ \ \quad L=\tfrac{{\bar{L}}}{\lambda }, \quad U_{0}=\dfrac{{\bar{U}}_{0}}{\text{ v }},\\ h&=\dfrac{{\bar{H}}}{r_{t}},\quad p=\dfrac{{\bar{P}}2\pi r_{t}^{2}}{\text{ v }\mu _{c} \lambda }, \qquad \quad \ \textbf{S}^{k}= \dfrac{\bar{\textbf{S}}^{k}r_{t}}{\text{ v }\mu _{c}}, \quad \quad \ \xi =\dfrac{2\pi r_{t}^{2}{\bar{\xi }}}{\text{ v }\mu _{c}}, \quad t_{rc}=\tfrac{2\pi \text{ v } {\bar{t}}_{rc}}{\lambda }. \end{aligned} \end{aligned}$$

Equations (4)–(8) after making use of dimensionless transformations (17) and the assumption of long wavelength approximation give rise to

*Peripheral fluid layer:*

18 $$\begin{aligned} \dfrac{\partial }{\partial {r}}\left( {r}{u}_{p}\right)&=-{r}\dfrac{\partial {w}_{p}}{\partial {z}}, \end{aligned}$$

19 $$\begin{aligned} \dfrac{1}{{r}}\dfrac{\partial }{\partial {r}}\left( {r}{S}_{{z}{r}}^{p}\right) -m\dfrac{\partial w_{p}}{\partial r}&= \dfrac{dp}{dz}, \end{aligned}$$

20 $$\begin{aligned} S_{zr}^{p}&=\dfrac{1}{\Lambda }\dfrac{\partial w_{p}}{\partial r}. \end{aligned}$$

*Core fluid layer:*

21 $$\begin{aligned} \dfrac{\partial }{\partial {r}}\left( {r}{u}_{c}\right)&=-{r}\dfrac{\partial {w}_{c}}{\partial {z}}, \end{aligned}$$

22 $$\begin{aligned} \dfrac{1}{{r}}\dfrac{\partial }{\partial {r}}\left( {r}{S}_{{z}{r}}^{c}\right)&= \dfrac{dp}{dz}, \end{aligned}$$

23 $$\begin{aligned} S_{zr}^{c}&= \dfrac{1}{\mu \Gamma }\dfrac{\partial w_{c}}{\partial r}, \end{aligned}$$

where we denote $Re=\dfrac{\rho _{c}\text{ v }r_{t}}{\mu _{c}}$ for Reynolds number, $\mu =\dfrac{\mu _{p}}{\mu _{c}}$ for effective viscosity, ${\bar{U}}_{0}$ for constant secretion of fluid from the permeable tube surface in the peripheral region, $\Lambda =\dfrac{1+\lambda _{1}^{p}}{\mu }$, $\Gamma =\dfrac{1+\lambda _{1}^{c}}{\mu }$ and $m=ReU_{0}$.

The utilization of the dimensionless transformations defined in Eq. (17) leads to the dimensionless forms of the peripheral travelling wave (1) and core travelling wave (2):

24 $$\begin{aligned} h(z)=1+\phi \sin \left( z\right) +\epsilon \phi \cos \left( \kappa z\right) \sin \left( z\right) , \end{aligned}$$

25 $$\begin{aligned} h_{1}(z)=1+\phi _{1} \sin \left( z\right) +\epsilon _{1} \phi _{1} \cos \left( \kappa z\right) \sin \left( z\right) , \end{aligned}$$

in which we denote $\phi =\dfrac{b}{r_{t}}$ for amplitude ratio and $\epsilon =\dfrac{2\pi A}{\lambda }$ for metachronal wave parameter of the peripheral travelling wave while $\phi _{1}=\tfrac{b_{1}}{r_{t_{1}}}$ for amplitude ratio and $\epsilon _{1}=\dfrac{2\pi A_{1}}{\lambda }$ for metachronal wave parameter of the core travelling wave.

Utilizing dimensionless transformations (17) into boundary conditions (9)–(16) respectively, we get

26 $$\begin{aligned} \dfrac{dp(0)}{dz}=-\xi . \end{aligned}$$

At $$r=0$$

27 $$\begin{aligned} S^{c}_{zr}\left( 0, z\right)&=0, \end{aligned}$$

28 $$\begin{aligned} u_{c} \left( 0, z\right)&=0. \end{aligned}$$

At $$r=h_{1}$$

29 $$\begin{aligned} w_{p}\left( h_{1}, z\right)&=w_{c}\left( h_{1}, z\right) , \end{aligned}$$

30 $$\begin{aligned} u_{p}\left( h_{1}, z\right)&=u_{c}\left( h_{1}, z\right) , \end{aligned}$$

31 $$\begin{aligned} S^{p}_{zr}\left( h_{1},z\right)&=S^{c}_{zr}\left( h_{1}, z\right) . \end{aligned}$$

At $$r=h$$

32 $$\begin{aligned} w_{p}\left( h, z\right)&=-1-\dfrac{\sqrt{Da}}{\beta }\dfrac{\partial }{\partial r}w_{p}(h,z), \end{aligned}$$

33 $$\begin{aligned} u_{p}\left( h, z\right)&=\dfrac{d h}{dz}, \end{aligned}$$

where $Da=\frac{K}{r_{t}^{2}}$ is the Darcy number.

## Solution of the problem

Solving Eq. (19) with the help of Eq. (20) whereas Eq. (22) with the help of Eq. (23) and then in turn utilising the boundary conditions (27), (29), (31) and (32), we acquire closed-form solutions of the form:

34 $$\begin{aligned} w_{p}(r,z)&=-1-\dfrac{1}{m^{2}\Lambda }\dfrac{dp}{dz}\left[ \left( ln r+m\Lambda r\right) -m^{2}\Lambda Ei(m\Lambda r)F_{1}\left( z\right) -m^{2}\Lambda F_{2}\left( z\right) \right] , \end{aligned}$$

35 $$\begin{aligned} w_{c}(r,z)&=-1+\dfrac{1}{4}\dfrac{dp}{dz}\left[ \Gamma \Lambda r^{2} +4F_{3}\left( z\right) \right] , \end{aligned}$$

where *Ei* denote for the exponential integral function and

$$\begin{aligned} F_{1}\left( z\right)&=\dfrac{e^{m\Lambda h_{1}}}{2m^{2}\Lambda }\left[ 2+2m \Lambda h_{1}+m^{2} \Lambda ^{2} h_{1}^{2}\right] , \\ F_{2}\left( z\right)&=\dfrac{1}{m^{2}\beta \Lambda h}\left[ m\beta \Lambda h^{2 }+\sqrt{Da}\left( 1+m \Lambda h\right) +\beta h \ ln h\right] -\dfrac{e^{m\Lambda h_{1}}}{2m^{2} \Lambda \beta h} \left[ \left( \sqrt{Da} \ e^{m \Lambda h}\right. \right. \\&\quad +\left. \left. \beta hEi(m\Lambda h)\right) \left( 2+2m \Lambda h_{1} +m^{2} \Lambda ^{2} h_{1}^{2}\right) \right] , \\ F_{3}\left( z\right)&=\dfrac{1}{m^{2}\beta \Lambda h}\left[ m\beta \Lambda h^{2}+\sqrt{Da}\left( 1+m \Lambda h\right) +\beta h \ ln h\right] -\dfrac{e^{m\Lambda h_{1}}}{2m^{2} \Lambda \beta h}\left[ \left( \sqrt{Da} \ e^{m \Lambda h}\right. \right. \\&\quad +\left. \left. \beta hEi(m\Lambda h)\right) \left( 2+2m \Lambda h_{1}+m^{2} \Lambda ^{2} h_{1}^{2}\right) \right] -\dfrac{1}{4m^{2}\Lambda }\left[ m\Lambda h\left( 4+m \Gamma \mu h\right) +4 ln h\right] \\&\quad +\dfrac{Ei(m \Lambda h)e^{m\Lambda h_{1}}}{2m^{2}\Lambda }\left[ 2+2m \Lambda h_{1}+m^{2} \Lambda ^{2} h_{1}^{2}\right] . \end{aligned}$$

Making use of Eq. (34) into Eq. (18) and Eq. (35) into Eq. (21) and then in turn invoking the respective boundary condition given in (28) and (33), we obtain the expressions for radial velocity:

36 $$\begin{aligned}u_{p}(r,z)&=\dfrac{1}{12m^{2}\Lambda^{2}}\dfrac{d}{dz}\dfrac{1}{r}\dfrac{dp}{dz}\left[\Lambda\left\{6\left(r^{2}lnr-h^{2}lnh\right)-3\left(r^{2}-h^{2}\right)+4m\Lambda\left(r^{3}-h^{3}\right)\right\}\right.\nonumber\\&-6\left.\left\{e^{m\Lambda r}\left(1-m\Lambda r\right)-e^{m\Lambda h}\left(1-m\Lambda h\right)+m^{2}\Lambda^{2}\left(r^{2}Ei(m\Lambda r)-h^{2}Ei(m\Lambda h)\right)\right\}\right.\nonumber\\&\times\left.F_{1}(z)-6m^{2}\Lambda^{2}\left(r^{2}-h^{2}\right)F_{2}(z)\right]-\dfrac{h}{r}\dfrac{dh}{dz},\end{aligned}$$

37 $$\begin{aligned} u_{c}(r,z)&=-\dfrac{1}{16}\dfrac{d}{dz}\dfrac{1}{r}\dfrac{dp}{dz}\left[ \Gamma \mu r^{4}+8r^{2}F_{3}(z)\right] . \end{aligned}$$

When we utilized Eqs. (36) and (37) into boundary condition (30) and then in turn for pressure gradient $\frac{dp}{dz}$ upon solving the resulting differential equation along with condition of pressure gradient at the permeable tube entrance (26), finally we arrive at

38 $$\begin{aligned} \frac{dp}{dz}=-\dfrac{\xi G(0)-24m^{2}\Lambda ^{2}\left( h^{2}-1\right) }{G(z)}, \end{aligned}$$

where

$$\begin{aligned} G\left( z\right)&=4\Lambda \left[ 6\left( h_{1}^{2}lnh_{1}-h^{2}lnh\right) -3\left( h_{1}^{2}-h^{2}\right) +4m\Lambda \left( h_{1}^{3}-h^{3}\right) \right] -24\left[ e^{m\Lambda h_{1}}\right. \\&\quad \times \left. \left( 1-m\Lambda h_{1}\right) -e^{m\Lambda h}\left( 1-m\Lambda h\right) +m^{2}\Lambda ^{2}\left( h_{1}^{2}Ei(m\Lambda h_{1})-h^{2}Ei(m\Lambda h)\right) \right] F_{1}(z)\\&\quad -24m^{2}\Lambda ^{2}\left( h_{1}^{2}-h^{2}\right) F_{2}(z)+ 3m^{2}\Lambda ^{2}\left[ \Gamma \mu h_{1}^{4}+8h_{1}^{2}F_{3}(z)\right] , \\ G\left( 0\right)&=4\Lambda \left[ 6\gamma ^{2}ln\gamma -3\left( \gamma ^{2}-1\right) +4m\Lambda \left( \gamma ^{3}-1\right) \right] -24\left[ e^{m\Lambda \gamma }\left( 1-m\Lambda \gamma \right) -e^{m\Lambda }\right. \\&\quad \times \left. \left( 1-m\Lambda \right) +m^{2}\Lambda ^{2}\left( \gamma ^{2} Ei(m\Lambda \gamma )-Ei(m\Lambda )\right) \right] F_{1}(0) -24m^{2}\Lambda ^{2}\left( \gamma ^{2}-1\right) F_{2}(0)\\&\quad +3m^{2}\Lambda ^{2}\left[ \Gamma \mu \gamma ^{4}+8\gamma ^{2}F_{3}(0)\right] , \end{aligned}$$

in which

$$\begin{aligned} F_{1}\left( 0\right)&=\dfrac{e^{m\Lambda \gamma }}{2m^{2}\Lambda }\left[ 2+2m \Lambda \gamma +m^{2} \Lambda ^{2} \gamma ^{2}\right] , \\ F_{2}\left( 0\right)&=\dfrac{1}{m^{2}\beta \Lambda }\left[ m\beta \Lambda +\sqrt{Da}\left( 1+m \Lambda \right) \right] -\dfrac{e^{m\Lambda \gamma }}{2m^{2} \Lambda \beta }\left[ \left( \sqrt{Da} \ e^{m \Lambda } +\beta Ei(m\Lambda )\right) \right. \\&\quad \times \left. \left( 2+2m \Lambda \gamma +m^{2} \Lambda ^{2} \gamma ^{2}\right) \right] , \\ F_{3}\left( 0\right)&=\dfrac{1}{m^{2}\beta \Lambda }\left[ m\beta \Lambda +\sqrt{Da}\left( 1+m \Lambda \right) \right] -\dfrac{e^{m\Lambda \gamma }}{2m^{2} \Lambda \beta }\left[ \left( \sqrt{Da} \ e^{m \Lambda } +\beta Ei(m\Lambda )\right) \right. \\&\quad \times \left. \left( 2+2m \Lambda \gamma +m^{2} \Lambda ^{2} \gamma ^{2}\right) \right] -\dfrac{1}{4m^{2}\Lambda }\left[ m\Lambda \left( 4+m \Gamma \mu \right) \right] +\dfrac{Ei(m \Lambda h)e^{m\Lambda h_{1}}}{2m^{2}\Lambda }\\&\quad \times \left[ 2+2m \Lambda \gamma +m^{2} \Lambda ^{2} \gamma ^{2}\right] . \end{aligned}$$

We define a dimensionless formula for the pressure difference over wavelength in the following way

39 $$\begin{aligned} \Delta p_{\lambda }=\int _{0}^{2\pi }\dfrac{dp}{dz} \ dz. \end{aligned}$$

Equation (39) with the aid of (38), can be employed to numerically integrate and derive the pressure difference over wavelength $\Delta p_{\lambda }$.

Many signs of development are important for understanding how a growing embryo moves through the human fallopian tube. These include the number and size of blastomeres, the percentage of fragmentation, and the presence of multinucleated blastomeres. Now, our focus is to find the residue time that, in most cases, corresponds to a good-quality embryo. A good-quality embryo typically exhibits 68 cells with uniform blastomeres, showing no signs of fragmentation or multinucleation. Ashraf et al.^1–5^ termed this residue time as “appropriate residue time”. We take into account that these developmental changes take place in the core fluid layer in our present two-layered model. The core fluid layer moves within the peripheral fluid layer during peristaltic-ciliary flow. Thus, an appropriate residue time is necessary for the growing embryo to attain the proper and complete developmental milestones, as in a good-quality embryo. We define an appropriate residue time and express it in dimensionless form as follows

40 $$\begin{aligned} t_{rc}=\int _{0}^{L}\dfrac{1}{w_{c}(r,z)}dz. \end{aligned}$$

Utilizing core region axial velocity (35) into (40), invoking different values of $\phi$, $\epsilon$, $\xi$, $\kappa$, *Da*, $\beta$, $\lambda _{1}^{p}$, $\lambda _{1}^{c}$, $\mu$ and one fixed value of *r* in the resulting equation and then in turn performing numerical integration we will get the appropriate residue time.

Stream functions $\psi _{p}$ and $\psi _{c}$ in dimensionless form respectively for the peripheral fluid layer and core fluid layer in the moving reference frame are defined as

41 $$\begin{aligned} u_{p}\left( r,z\right)&=-r^{-1}\dfrac{\partial \psi _{p}}{\partial z}, \quad w_{p}\left( r,z\right) =r^{-1}\dfrac{\partial \psi _{p}}{\partial r}, \\ u_{c}\left( r,z\right)&=-r^{-1}\dfrac{\partial \psi _{c}}{\partial z}, \quad w_{c}\left( r,z\right) =r^{-1}\dfrac{\partial \psi _{c}}{\partial r}. \end{aligned}$$

Zero value of streamline at the midplane and condition of streamline at the interface of peripheral fluid layer and core fluid layer, respectively are given by

42 $$\begin{aligned} \psi _{c}\left( 0,z\right)&=0, \end{aligned}$$

43 $$\begin{aligned} \psi _{p}\left( h_{1},z\right)&=\psi _{c}\left( h_{1},z\right) . \end{aligned}$$

Using axial velocity components from Eqs. (34) and (35) into Eq. (41) and then in turn utilizing the zero value of streamline (42) and condition of streamline at the interface (43), we finally arrive at

44 $$\begin{aligned} \psi _{p}(r,z)&=-\dfrac{1}{12m^{2}\Lambda ^{2}}\dfrac{dp}{dz} \left[ \Lambda \left\{ 6\left( r^{2}lnr-h_{1}^{2}lnh_{1}\right) -3\left( r^{2}-h_{1}^{2} \right) +4m\Lambda \left( r^{3}-h_{1}^{3}\right) \right\} \right. \nonumber \\&\quad -6\left. \left\{ e^{m\Lambda r}\left( 1-m\Lambda r\right) -e^{m\Lambda h_{1}}\left( 1-m\Lambda h_{1}\right) +m^{2}\Lambda ^{2}\left( r^{2}Ei(m\Lambda r)-h_{1}^{2}Ei(m\Lambda h_{1})\right) \right\} \right. \nonumber \\&\quad \times \left. F_{1}(z)-6m^{2}\Lambda ^{2} \left( r^{2}-h_{1}^{2}\right) F_{2}(z)\right] -\dfrac{r^{2}}{2}+\dfrac{1}{16} \dfrac{dp}{dz}\left[ \Gamma \mu h_{1}^{4}+8h_{1}^{2}F_{3}(z)\right] , \end{aligned}$$

45 $$\begin{aligned} \psi _{c}(r,z)&=\dfrac{1}{16} \dfrac{dp}{dz}\left[ \Gamma \mu r^{4}+8r^{2}F_{3}(z)\right] -\dfrac{r^{2}}{2}. \end{aligned}$$

## Results

**Figure 2:**
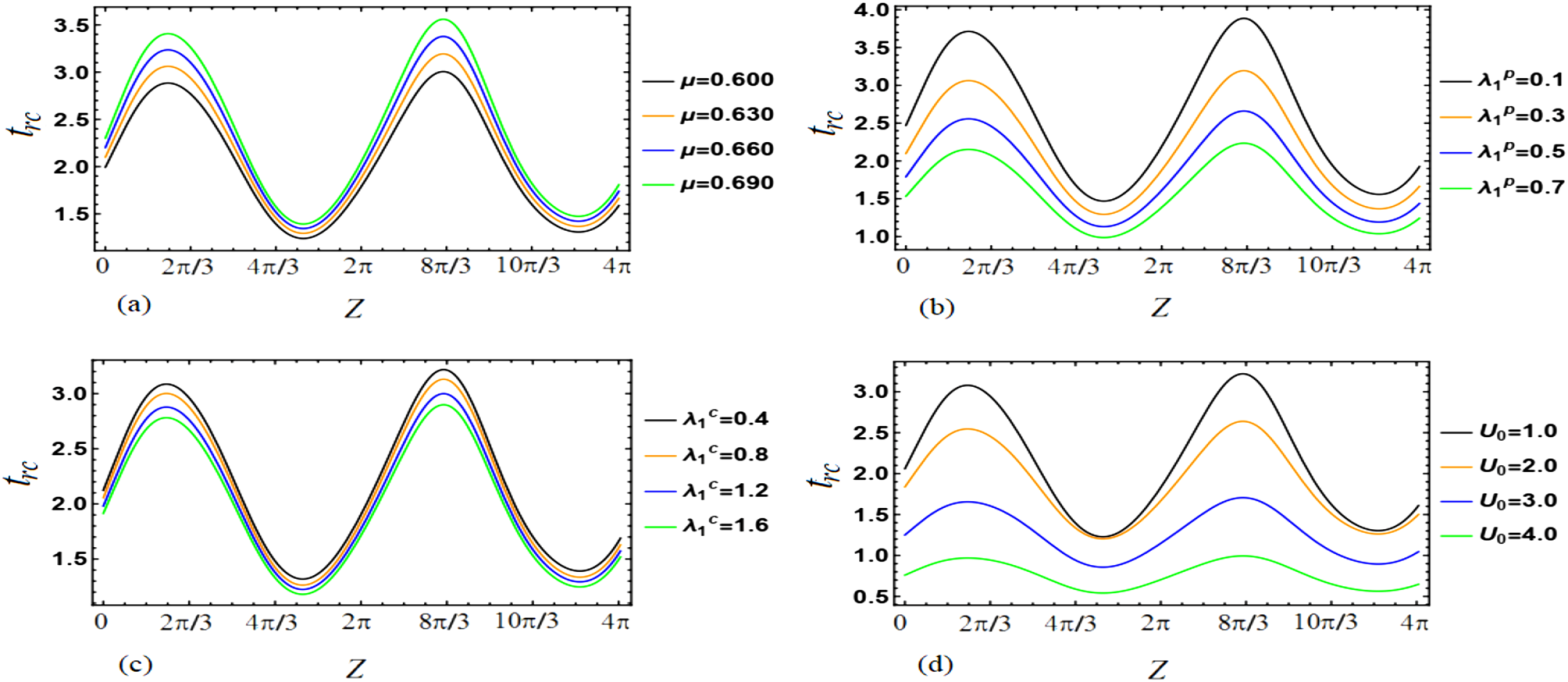
Variations in $t_{rc}$ as a function of *z* for different values of the parameters (**a**) $\mu$, (**b**) $\lambda _{1p}$, (**c**) $\lambda _{1c}$, and (**d**) $U_0$.

**Figure 3:**
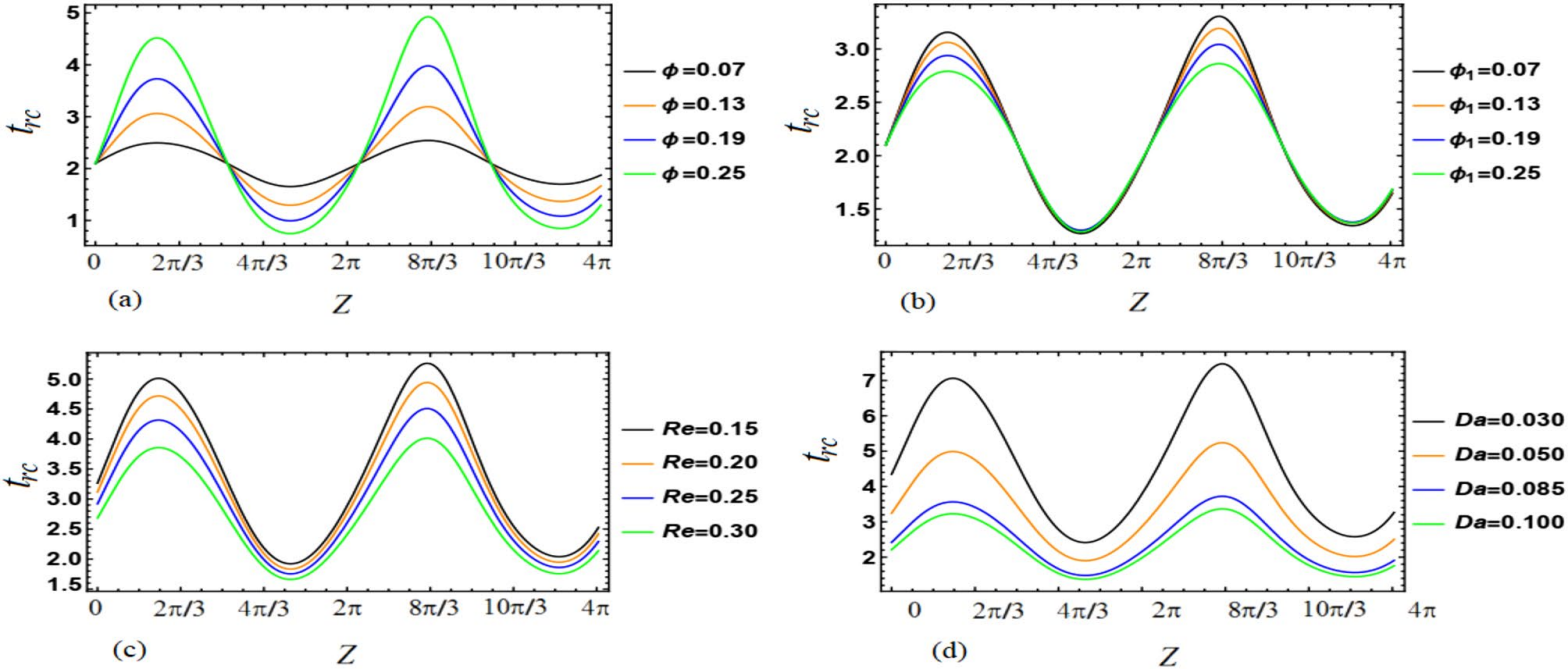
Variations in $t_{rc}$ as a function of *z* for different values of the parameters (**a**) $\phi$, (**b**) $\phi _{1}$, (**c**) *Re* and (**d**) *Da*.

**Figure 4:**
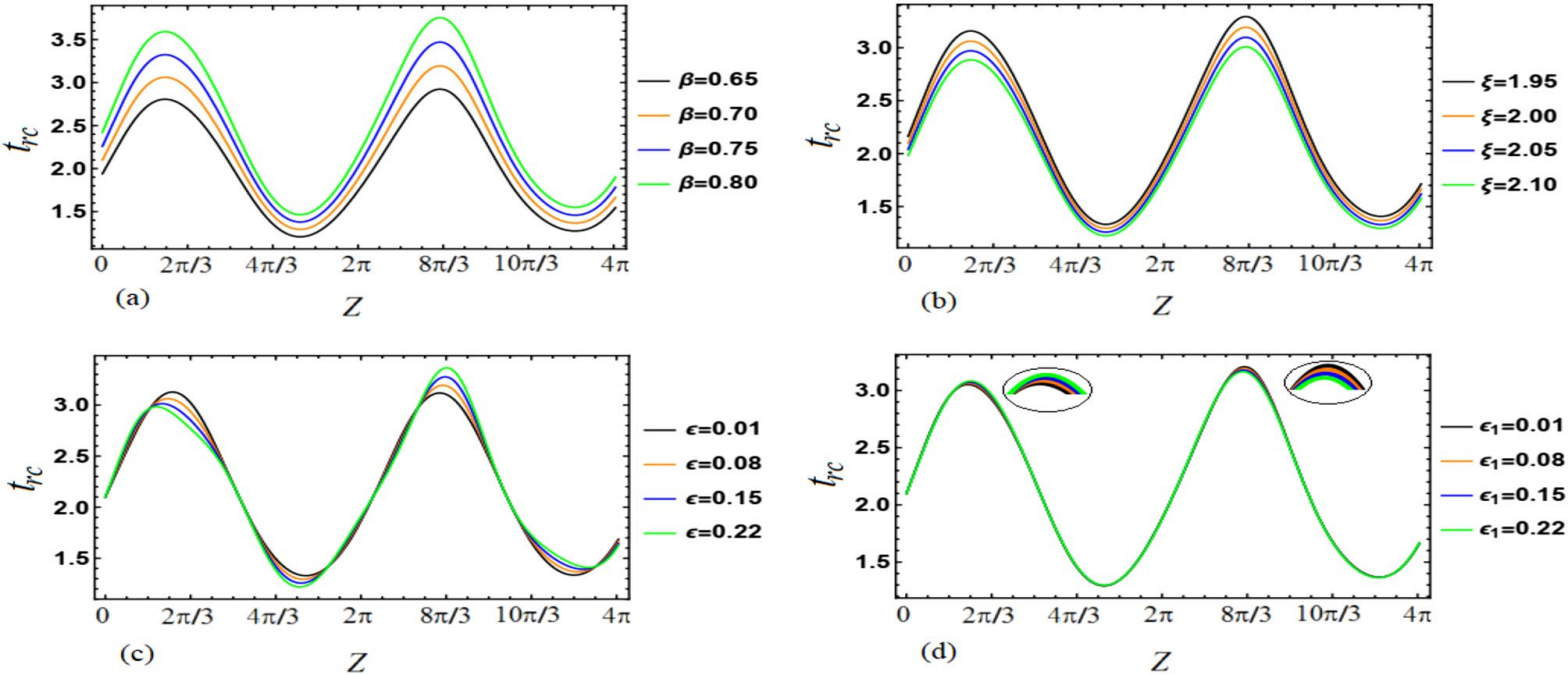
Variations in $t_{rc}$ as a function of *z* for different values of the parameters (**a**) $\beta$, (**b**) $\xi$, (**c**) $\epsilon$ and (**d**) $\epsilon _{1}$.

**Figure 5:**
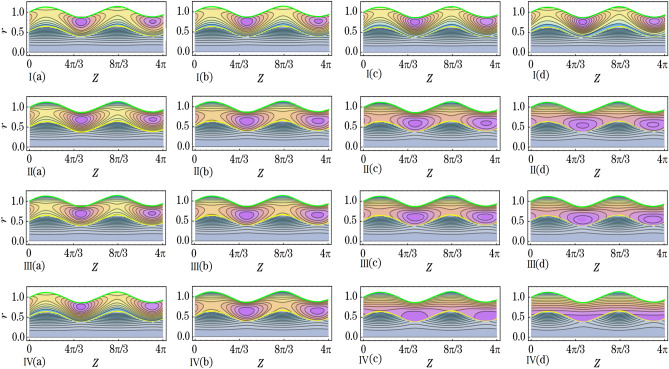
The effects of (**I**) $\mu$ ((a) 0.600, (b) 0.630, (c) 0.660 and (d) 0.690), (**II**) $\lambda _{1}^{p}$ ((a) 0.1, (b) 0.3, (c) 0.5 and (d) 0.7), (**III**) $\lambda _{1}^{c}$ ((a) 0.4, (b) 0.8, (c) 1.2 and (d) 1.6) and (**IV**) $U_{0}$ ((a) 1.0, (b) 2.0, (c) 3.0 and (d) 4.0) on size of the trapped boluses.

**Figure 6:**
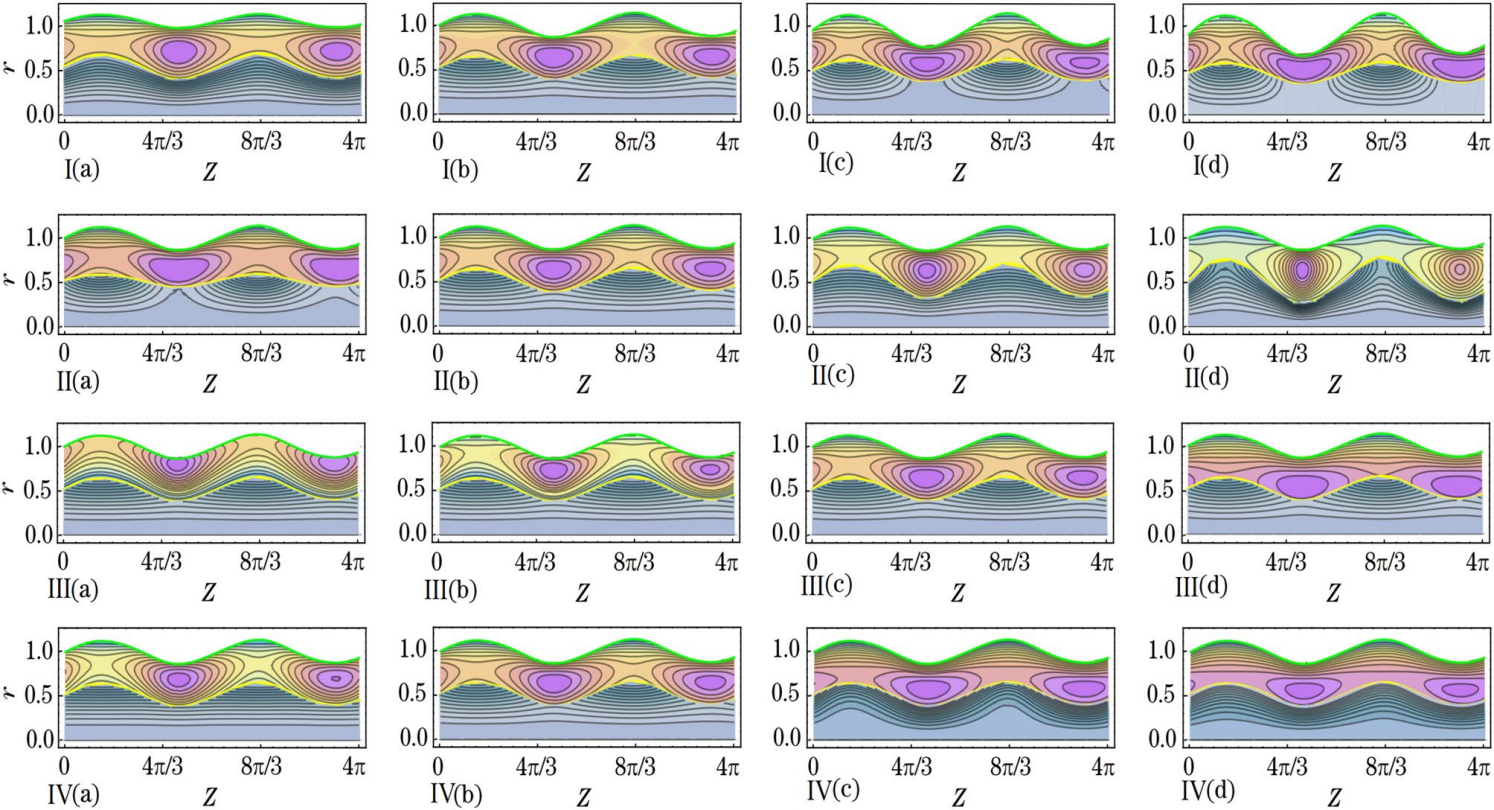
The effects of (**I**) $\phi$ ((a) 0.07, (b) 0.13, (c) 0.19 and (d) 0.25), (**II**) $\phi _{1}$ ((a) 0.07, (b) 0.13, (c) 0.19 and (d) 0.25), (**III**) *Re* ((a) 0.15, (b) 0.20, (c) 0.25 and (d) 0.30) and (**IV**) *Da* ((a) 0.030, (b) 0.050, (c) 0.085 and (d) 0.100) on size of the trapped boluses.

**Figure 7:**
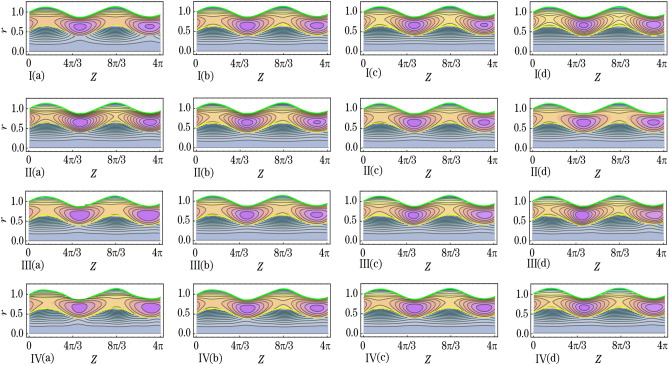
The effects of (**I**) $\beta$ ((a) 0.65, (b) 0.70, (c) 0.75 and (d) 0.80), (**II**) $\xi$ ((a) 1.95, (b) 2.0, (c) 2.5 and (d) 3.0), (**III**) $\epsilon$ ((a) 0.01, (b) 0.08, (c) 0.15 and (d) 0.22) and (**IV**) $\epsilon _{1}$ ((a) 0.01, (b) 0.08, (c) 0.15 and (d) 0.22) on size of the trapped boluses.

This section aims to provide a theoretical analysis of the impact of geometric parameters, fluid model parameters, and Reynolds number (*Re*) on the flow variables of the size of trapped boluses and appropriate residue time in the context of human embryo transport in the fallopian tube^1–5^. Geometric parameters include metachronal wave parameters ($\epsilon$, $\epsilon _{1}$), amplitude ratios ($\phi$, $\phi _{1}$), Darcy number *Da*, slip parameter $\beta$, constant secretion rate $U_{0}$, and condition of pressure gradient at the permeable tube entrance $\xi$. Fluid model parameters, on the other hand, include the effective viscosity $\mu$ and ratios of relaxation time to retardation time $(\lambda _{1}^{p}$, $\lambda _{1}^{c})$. We plotted graphs (see Figs. 2, 3, 4, 5, 6, 7) in order to observe the quantitative effects of $\mu$, $\lambda _{1}^{p}$, $\lambda _{1}^{c}$, $U_{0}$, $\phi$, $\phi _{1}$, *Re*, *Da*, $\beta$, $\xi$, $\epsilon$ and $\epsilon _{1}$ on aforesaid flow variables.

### Appropriate residue time

To investigate the impact of various parameters on appropriate residue time, we generated plots for a frontal cross-section (0.35, *z*), as shown in Figs. 2, 3 and 4. Our analysis considered factors such as $\mu$, $\lambda _{1}^{p}$, $\lambda _{1}^{c}$, $U_{0}$, $\phi$, $\phi _{1}$, *Re*, *Da*, $\beta$, $\xi$, $\epsilon$, and $\epsilon _{1}$ (as defined in Eq. (42)). From Fig. 2a, it can be inferred that an increase in $\mu$ results in an increase in appropriate residue time. Since $\mu$ is the ratio of fluid viscosities $\mu _{p}$ and $\mu _{c}$, this suggests a direct relationship between $\mu _{p}$ and residue time, and an inverse relationship between $\mu _{c}$ and residue time. Figure 2b,c demonstrate that appropriate residue time increases with an increase in $\lambda _{1}^{p}$ and $\lambda _{1}^{c}$, with the impact of $\lambda _{1}^{p}$ being more prominent than that of $\lambda _{1}^{c}$. Figure 2d reveals that an increase in $U_{0}$ leads to a decrease in residue time.

Figure 3a,b indicate that appropriate residue time increases with an increase in $\phi$, but decreases with an increase in $\phi _{1}$. Here, $\phi =\tfrac{b}{r_{t}}$ and $\phi _{1}=\tfrac{b_{1}}{r_{t_{1}}}$, where *b* and $b_{1}$ represent the amplitude of peripheral and core sinusoidal waves, respectively. This suggests that residue time increases with the amplitude of a peripheral sinusoidal wave and decreases with the amplitude of a core sinusoidal wave. Moreover, Fig. 3c shows that an increase in Reynolds number (*Re*) results in a decrease in appropriate residue time. Similarly, the impact of *Da* on residue time delineate a decrease, as shown in Fig. 3d. In this case, *Da* is related to the permeability of the porous medium, represented by the parameter *K*. A higher value of *K* means that the opening of the pores in goblet cells is larger during the exocytosis process, resulting in the secretion of a greater quantity of fluid. Therefore, a larger quantity of fluid secreted leads to a lesser appropriate residue time.

Figure 4a depicts that appropriate residue time varies directly with $\beta$. When $\xi$ increases then appropriate residue time decreases as can be noted from Fig. 4b. In other words, one may reveal that more pressure gradient at the permeable tube entrance lesser the residue time. Figure 4c depicts overall increment in appropriate residue time with increasing $\epsilon$ whilst Fig. 4d depicts overall diminishment in appropriate residue time with increasing $\epsilon _{1}$. $\epsilon =\tfrac{2\pi A}{\lambda }$ and $\epsilon _{1}=\tfrac{2\pi A_{1}}{\lambda }$ disclose that $\epsilon$ varies directly with *A* (amplitude of peripheral metachronal wave) whereas $\epsilon _{1}$ varies directly with $A_{1}$ (amplitude of core metachronal wave). Both the amplitudes of the peripheral metachronal wave and core metachronal wave have vice versa impact on appropriate residue time. When there is an increment in amplitudes of both the peripheral sinusoidal and metachronal waves then appropriate residue time also increments. On the other hand, with the increment in amplitudes of both the core sinusoidal and metachronal waves, the appropriate residue time decreases.

### Trapped bolus

We prepared Figs. 5, 6 and 7 to infer the effects of the variation of $\mu$, $\lambda _{1}^{p}$, $\lambda _{1}^{c}$, $U_{0}$, $\phi$, $\phi _{1}$, *Re*, *Da*, $\beta$, $\xi$, $\epsilon$, and $\epsilon _{1}$ on bolus size of secreted out fluid trapped in the peripheral fluid layer. Between the tube surface and peripheral fluid layer-core fluid layer interface elliptically shaped fluid boluses are formed where the tube has a local maximum diameter. As the diameter reaches a local minimum, the trapped boluses gradually decrease in size until they disappear completely. This phenomenon is visualized through the use of streamline plots (refer to Figs. 5, 6, 7). Streamlines in the peripheral fluid layer split and close to form boluses that move as a whole with the wave. In terms of the density of streamlines, we describe the size of the trapped boluses. It is noted from Fig. 5I that bolus size enhances by the increase of $\mu$. Bolus size enhances with the increase of $\mu _{p}$. On the other hand, it gets a decrease in size with the increase of $\mu _{c}$. Figure 5II,III delineate that bolus gets a reduction in size with an increase in the $\lambda _{1}^{p}$ and $\lambda _{1}^{c}$. But the impact of $\lambda _{1}^{p}$ is more prominent on bolus size than that of the $\lambda _{1}^{c}$. Figure 5IV depicts that bolus size gets shorter when values of $U_{0}$ are increased from 1.0 to 2.0 and eventually disappear when values of $U_{0}$ are increased from 3.0 to 4.0.

Figure 6I,II elucidate that $\phi$ and $\phi _{1}$ have likewise effects on bolus size. With an increase in the $\phi$ and $\phi _{1}$ bolus size enlarges. Specifically saying one infers that with the increase in $\phi$ the length of the bolus increases whereas the width of the bolus increases with the increase in $\phi _{1}$. When amplitudes of both the peripheral sinusoidal wave and core sinusoidal wave are increased then bolus in size gets enlarged. Figure 6III which depicts the effect of *Re* renders that when the values of *Re* are increased then bolus size get diminishes. In Fig. 6IV we delineate that by the increment of *Da* the bolus size get diminishes likewise the impact of *Re*. Bolus size increases with an increase in the permeability of the porous media. With that stated one can reveal the fact that the larger the opening of the pores of goblet cells more amount of fluid will be secreted that in turn causes an increment in bolus size. Boluses also shift their positions away from the tube surface. From Fig. 7I it is perceived that when the values of $\beta$ are increased then bolus size get enhances. By the increase of $\xi$ the bolus size get enhances as can be seen through Fig. 7II. Through Fig. 7III,IV, we noted that by the increase of $\epsilon$ and $\epsilon _{1}$ the bolus size gets larger. Both the amplitudes of the peripheral metachronal wave and core metachronal wave have a similar impact on bolus size. Moreover, one may link this size increment of bolus with the increase in amplitudes of the sinusoidal wave and the metachronal wave.

### Summary of results

Effects of various emerged parameters on appropriate residue time and trapped boluses size have been considered, including geometric parameters (metachronal wave parameters ($\epsilon$, $\epsilon _{1}$), amplitude ratios ($\phi$, $\phi _{1}$), Darcy number *Da*, slip parameter $\beta$, constant secretion rate $U_{0}$ and condition of pressure gradient at the permeable tube entrance $\xi$), fluid model parameters (effective viscosity $\mu$ and ratios of relaxation time to retardation time $(\lambda _{1}^{p}$, $\lambda _{1}^{c})$), and Reynolds number *Re*. We summarized the important results as follows:

- Appropriate residue time increases by the increase of $\phi$, $\beta$, $\epsilon$ and $\mu$ whereas it decreases by the increase of $\xi$, $\phi _{1}$, $\epsilon _{1}$, *Da*, $\lambda _{1}^{p}$ and $\lambda _{1}^{c}$, $U_{0}$ and *Re*. Greater the constant secretion rate lesser the residue time of core fluid layer.
- Trapped boluses size enlarges by the increase of $\phi$, $\beta$, $\epsilon$, $\phi _{1}$, $\epsilon _{1}$ and $\mu$ while their size reduces by the increase of $\xi$, *Da*, $\lambda _{1}^{p}$ and $\lambda _{1}^{c}$, $U_{0}$ and *Re*. The trapped boluses finally disappear after the constant secretion velocity exceeds 3.0. When smaller trapped boluses form within the peripheral fluid layer, the core fluid layer moves smoothly in the axial direction.

A two-layered biomechanical model is a more effective approach to analyze the transport of a growing embryo in the human fallopian tube, due to the distinct differences in viscosity and density between the continuously secreting fluid and the growing embryo.

## Discussion

### Theoretical analysis

A biomechanical model that incorporates a finite permeable tube consisting of two layers, where the Jeffrey fluid model characterizes the viscoelastic properties of the growing embryo and secreted fluid was developed. The finite permeable tube considers three regions of the fallopian tube: ampullar, isthmus and intramural. With a pressure gradient of $-\xi$, the ovum gets into the ampullar region. An embryo is formed when the ovum and spermatozoon fuse. From the ampullar region to the intramural region of the fallopian tube, the growing embryo within the continuously secreting fluid is subsequently transported by the peristalsis-cilia. The flow variables that affect how an embryo grows were looked at. These are the appropriate residue time ($t_{rc}$) and stream functions ($\psi _{c}$ and $\psi _{p}$) for different sets of geometric parameters, fluid model parameters, and pressure gradient at the permeable tube entrance.

The current biomechanical model becomes applicable when considering the effects of varying geometric parameters, fluid model parameters, and pressure gradient on the transport of the growing embryo within the human fallopian tube. The transportation time of the growing embryo from the entrance of the ampullar region to the exit of the intramural region is called the appropriate residue time. It increases with the increase of $\phi$, $\beta$, $\epsilon$, and $\mu$, whereas it decreases with the increase of $\xi$, $\phi _{1}$, $\epsilon _{1}$, *Da*, $\lambda _{1}^{p}$ and $\lambda _{1}^{c}$, $U_{0}$, and *Re*. The reported appropriate residue time is about 34 days. Boluses of continuously secreting fluid from the goblet cells are trapped near the fallopian tube surface. The growing embryo moves within these trapped boluses. The size of these boluses gets reduced by the increase of $\xi$, *Da*, $\lambda _{1}^{p}$ and $\lambda _{1}^{c}$, $U_{0}$, and *Re*. On the other hand, their size enlarges with the increase of $\phi$, $\beta$, $\epsilon$, $\phi _{1}$, $\epsilon _{1}$, and $\mu$. Trapped boluses eventually disappear when values of constant secretion velocity are greater than 3.0. The growing embryo moves smoothly in the axial direction when trapped boluses of smaller size are formed near the tube surface.

LH, FSH, and prolactin control the release amount of $E_{2}$ and $P_{4}$. In turn, $E_{2}$ and $P_{4}$ through specific hormone receptors play an important role in the normal functioning of the smooth muscle cells, ciliary cells, and goblet cells. AMH could affect how the ovum is released and transported into the fallopian tubes. Thus, AMH can indirectly regulate the transport of a growing embryo. $E_{2}$ and prolactin regulates the level of TSH. There exists an inverse relationship between the $P_{4}$ and various factors, including the amplitudes of the peripheral sinusoidal and core sinusoidal waves, the amplitudes of the peripheral metachronal and core metachronal waves, and the amount of secreting fluid. In contrast, $E_{2}$ is directly proportional to these factors. Additionally, the pressure gradient at the entrance of the ampullar region has a direct relationship with $P_{4}$ and an inverse relationship with $E_{2}$^4,5^. The amplitudes of both the peripheral sinusoidal and metachronal waves are directly proportional to the appropriate residue time. On the other hand, the amplitudes of both the core sinusoidal and metachronal waves are inversely proportional to the appropriate residue time. There is a direct link between the size of the boluses and the amplitudes of the peripheral sinusoidal wave, the core sinusoidal wave, the peripheral metachronal wave, and the core metachronal wave. With that said, one can now add that the appropriate residue time increases and boluses grow in size as more $E_2$ is released through the respective receptors. On the other hand, when more $P_4$ is released through the receptor, the appropriate residue time reduces and boluses shrink in size. When $E_{2}$ is released through its receptors in larger amounts than what is needed, goblet cells secrete more fluid continuously. Consequently, this particular circumstance results in the swelling of the fallopian tube due to the accumulation of fluid, ultimately resulting in the obstruction of the fallopian tube. In contrast, in instances where there is an excessive release of $P_{4}$ through the receptor, goblet cells do not secrete any fluid. This scenario may result in dryness within the fallopian tube. Therefore, the transport of the growing embryo is regulated by $P_{4}$ and $E_{2}$ directly, and LH, FSH, prolactin, AMH, and TSH indirectly. This regulation is achieved by controlling the amplitudes of both the sinusoidal and metachronal waves, maintaining continuous secretion of fluid through the goblet cells, and establishing a pressure gradient at the entrance of the ampullar region. By accounting for these variations in physical properties, a two-layered biomechanical model is a more effective approach to analyze the transport of a growing embryo in the human fallopian tube.

### Experimental analysis

**Figure 8:**
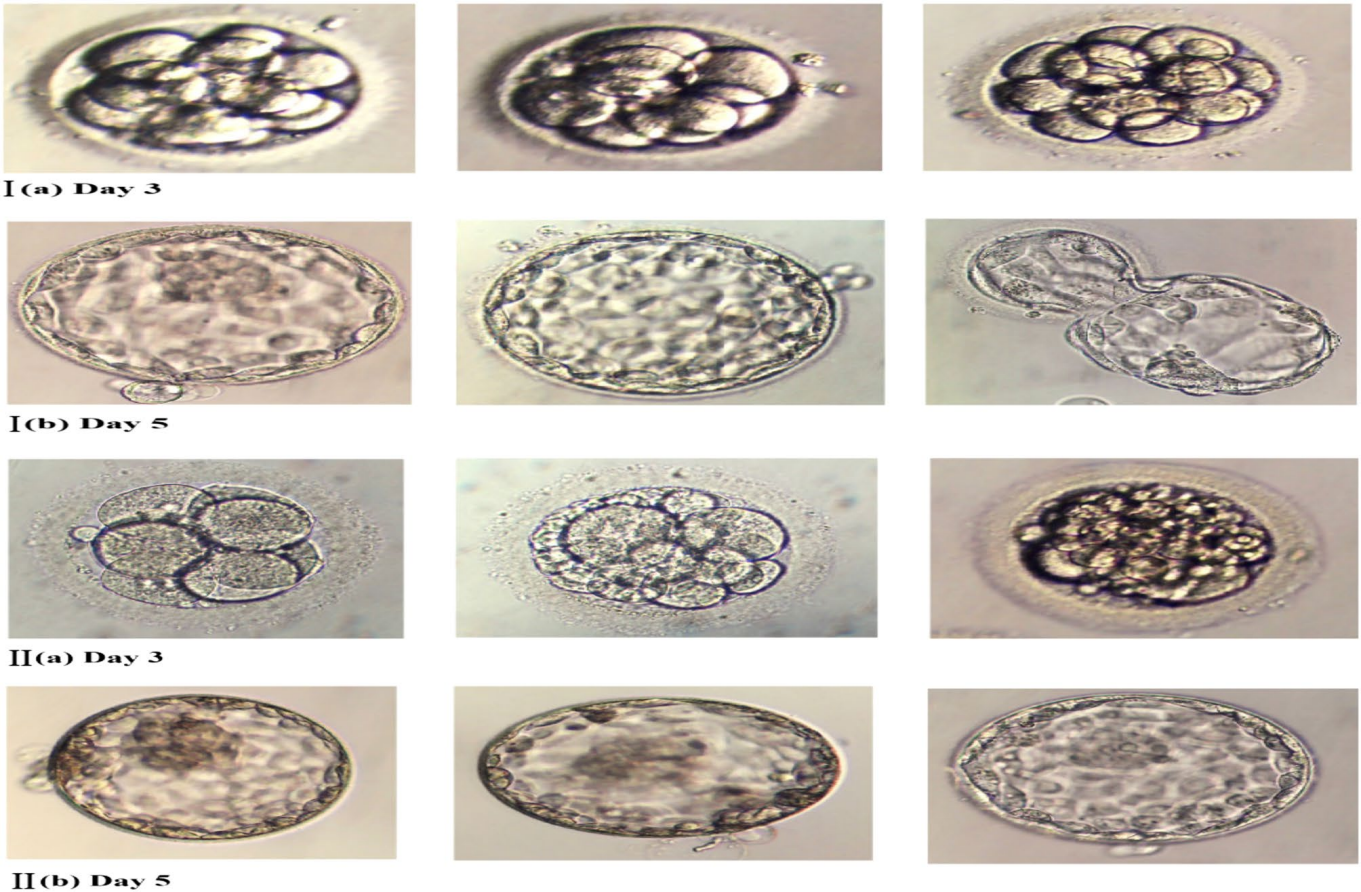
Phase contrast images of (**I**) good quality embryos and (**II**) bad quality embryos on day 3 and day 5 of in vitro culture.

This subsection aims to provide prospective experimental in vitro analysis of human embryo preimplantation development. In this regard, embryo phase contrast images have been taken in the laboratory (see Fig. 8). We evaluated the quality of the embryos on day 3 and day 5 of culture. Classified and allocated them into two groups according to the number and size of blastomeres, the percentage of fragmentation, and the presence of multinucleated blastomeres. In group 1, we took grade I embryos (good quality) with 6–8 cells, without fragmentation, and with equal-sized blastomeres with an absence of multinucleation (see Fig. 8I). In group 2, we took grade II embryos (bad quality) with $<6$ blastomeres and/or $>50\%$ fragmentation (see Fig. 8II).

We tabulate experimental numerical data for baseline hormones, type of infertility, outcome parameters, and pregnancy outcomes in Table 1. During the preimplantation phase, laboratory assessments were carried out on human embryos on days 3 and 5 of culture. According to our results, the mean age was $32.61 \pm 5.73$. In group 1 (good-quality embryo), out of 50 patients, $36 (52.2\%)$ had primary infertility and 14 (45.2%) had secondary infertility, and in group 2 (bad-quality embryo), 33 (47.8%) had primary infertility, and 17 (54.8%) had secondary infertility. The stimulation protocol in both groups was agonist and antagonist. In group 1, 24 (51.1%) patients received an agonist stimulation protocol, and 20 (51.3%) received antagonists. On the other hand, in group 2, 23 (48.9%) patients received agonist stimulation protocol, and 19 (48.7%) received antagonists.

In this table, we tabulated the baseline hormonal profile in mean and standard deviation. The mean body mass index (MBI) was the same at $28 \pm 5.62$ in both groups. The mean basal serum FSH concentration in both groups was $7.35 \pm 3.01$ and $7.67 \pm 4.01$. The mean LH was $5.24 \pm 2.99$ in group 1, and the mean LH was $7.87 \pm 10.59$ in group 2. In group 1, the mean prolactin was $15.54 \pm 9.29$. In group 2, the mean prolactin was $23.26 \pm 43.11$. The mean $E_{2}$ was $49.54\pm 29.11$, and $63.96 \pm 61.53$ in group 1 and group 2 respectively. In group 1, the mean AMH was $2.78 \pm 1.72$, and in group 2, the mean AMH was $3.42 \pm 3.88$. The level of TSH was $2.43 \pm 2.68$ and $1.73 \pm 1.08$ in both groups. There were no differences in the primary outcomes of the study on embryo quality with respect to the total number of oocytes obtained, fertilized oocytes, and the number of embryos transferred in both groups. However, the pregnancy outcomes were significantly better in the good quality embryo. Out of 50 patients, $26\, (76.5\%)$ were pregnant in group 1, and only $8\, (23.5\%)$ were in group 2.

These findings suggests that stimulation protocol (agonist and antagonist) and baseline hormones (*FSH*, *LH*, prolactin, $E_{2}$, *AMH* and *TSH*) play vital role in embryo preimplantation in vitro development. In cases where a female has undergone a stimulation protocol and her embryo has baseline hormonal values of good quality embryo, a properly developed embryo with complete mitotic divisions will be transported from the ampullar region to the intramural region of the human fallopian tube. In this case pregnancy outcomes will be positive. On the other hand, in the case where a female has not undergone a stimulation protocol and her embryo exhibits poor baseline hormonal values, successful transport through the fallopian tube may not occur due to incomplete or improper mitotic divisions. In this case pregnancy outcomes will be negative.

**Table 1:** Characteristics of different variables with respect to embryo quality.

Variables	Embryo quality	
	Good quality	Bad quality
No. of patients	50	50
Age (years)	32.61 ± 5.73	32.02± 5.74
Infertility diagnosis $[n (\%)]$		
Type of infertility		
Primary	36 (52.2)	33 (47.8)
Secondary	14 (45.2)	17 (54.8)
Stimulation protocol $[n (\%)]$		
Agonist	24 (51.1)	23 (48.9)
Antagonist	20 (51.3)	19 (48.7)
Baseline hormonal profile (mean ± S.D)		
BMI (kg/m$^{2}$)	28 ± 5.62	28.85 ± 5.48
FSH (mIU/ml)	7.35 ±3.01	7.67 ± 4.01
LH (IU/l)	5.24 ± 2.99	7.87 ± 10.59
Prolactin (ng/ml)	15.54 ± 9.2	23.26 ± 43.11
E2 (pg/ml)	49.54 ± 29.11	63.96 ± 61.53
AMH (ng/ml)	2.78 ± 1.72	3.42 ± 3.88
TSH (uIU/ml)	2.43 ± 2.68	1.73 ± 1.08
Outcome parameters (mean ± S.D)		
No. of total oocytes	10.72 $\pm$ 6.76	9.96 ± 5.51
No. of fertilized oocytes	4.74 ± 2.89	4.72 ± 3.54
No. of embryo transferred	1.78 ± 0.64	1.86 ± 0.75
Pregnancy outcome		
Positive	26 (76.5)	8 (23.5)
Negative	24 (36.4)	42 (63.6)

### Weaknesses and strengths of the model

This analysis provides a biomechanical model that contains a finite permeable tube with two layers, where the Jeffrey fluid model characterizes the viscoelastic nature of the growing embryo and continually secreting fluid.

#### Weaknesses of the model

The weaknesses of the proposed two-layered biomechanical model are of a modelling nature. The original human fallopian tube has a converging, narrow tube shape that extends from the ovary to the uterus. The human fallopian tube, which has a non-uniform cross-section, is modelled as a straight, permeable tube with a uniform cross-section. This tube has three regions: the ampullar, the isthmus, and the intramural. The mucus membrane of the fallopian tube is crowded with columnar epithelial cells: secretory goblet cells, basel cells, ciliary cells, and peg cells. The distribution of these epithelial cells varies along the tube. The mucus membrane of the considered straight, permeable tube is assumed to be crowded with uniformly distributed secretory goblet cells and ciliary cells only. The growing embryo is of a viscoelastic nature and spherical in shape, as it grows as a result of mitotic divisions. A growing embryo transport is not a fully developed peristaltic-ciliary flow. It is hypothesized that peristaltic-ciliary flow is fully developed.

#### Strengths of the model

The proposed two-layered biomechanical model’s strength is its ability to accurately depict the dynamics of a growing embryo and a continuously secreting fluid as two immiscible layers. This model is more suitable since it takes into consideration the different densities and viscosities of the embryo and secreting fluid. Through the use of the concept of permeable surfaces with injection and the consideration of a continuous secretion of viscoelastic fluid through the goblet cells, this model sheds light on the regulation of embryo transportation through both direct factors, like $P_4$ and $E_2$, and indirect factors, like LH, FSH, prolactin, AMH, and TSH.

## Concluding remarks

Prospective analysis that uses the two-layered biomechanical model of peristaltic-ciliary transport has been addressed. The Jeffrey fluid model has been used to characterize viscoelastic natured continuously secreting fluid in the peripheral fluid layer and fluid entering with some negative pressure gradient in the core fluid layer. The subsequent linear partial differential equations have been solved for closed-form solutions. Appropriate residue time of the core fluid layer and trapped boluses size of the peripheral fluid layer have been used as flow variables. Effects of various emerged parameters on these flow variables have been considered, including geometric parameters, fluid model parameters, constant secretion velocity, condition of pressure gradient at the permeable tube entrance and Reynolds number. An experimental in vitro analysis of human embryo preimplantation development has also been carried out in the laboratory. We summarized the important findings of the current biomechanical analysis as follows:

- The greater the constant secretion velocity, the lesser the residue time of the core fluid layer, and smaller-sized trapped boluses are formed in the peripheral fluid layer. Trapped boluses eventually disappear when values of constant secretion velocity are higher than 3.0. The core fluid layer moves smoothly in the axial direction when trapped boluses of smaller size are formed in the peripheral fluid layer.
- The amplitudes of the peripheral and core travelling waves and quantity of continuously secreting fluid are constraint directly by the $P_4$ and $E_2$, while indirectly constraint by LH, FSH, prolactin, AMH, and TSH.
- The pregnancy outcomes in the good quality embryo were significantly better than the bad quality embryo. 26 (76.5%) out of 50 patients were pregnant in group 1 with a good quality embryo, and only 8 (23.5%) out of 50 were in group 2 with a bad quality embryo.
- Baseline hormones *FSH*, *LH*, prolactin, $E_{2}$, *AMH* and *TSH* constraint the human embryo preimplantation development in in vitro.

The findings of the current analysis suggest that peristaltic-ciliary transport, consisting of two layers, is a more accurate representation of the way in which the growing human embryo is transported through the fallopian tube. Baseline hormones *FSH*, *LH*, prolactin, $E_{2}$, *AMH*, and *TSH* constraint the human embryo preimplantation development in in vitro as well as the motion of the growing embryo in the fallopian tube. In essence, this study contributes to our understanding of how baseline hormones control early developmental processes and the fallopian tube’s role in the transportation of human embryos.

## Data Availability

The data used to support the findings of this study are available from the corresponding author upon request.

## List of symbols

${\bar{t}}$: Instant of time (s)
$\text{ v }$: Wave speed (mm s$^{-1}$)
${\bar{\xi }}$: Pressure gradient at the permeable tube entrance (g mm$^{-2}$ s$^{2}$)
$\mu _{k}$: Dynamic viscosity (poise)
$\rho _{k}$: Constant density of the fluid (g mm$^{-3}$)
${\bar{S}}^{k}_{{\bar{R}}{\bar{R}}}, {\bar{S}}^{k}_{{\bar{R}}{\bar{Z}}}, {\bar{S}}^{k}_{{\bar{\theta }} {\bar{\theta }}}, {\bar{S}}^{k}_{{\bar{Z}}{\bar{R}}}, {\bar{S}}^{k}_{{\bar{Z}}{\bar{Z}}}$: Shear stresses (g mm$^{-1}$ s$^{-2}$)
$A,\ A_{1}$: Amplitudes of metachronal waves (mm)
$b,\ b_{1}$: Amplitudes of sinusoidal waves (mm)
$Ab,\ A_{1}b_{1}$: Maximum displacements of material points of ciliary cells (mm)
$r_{t}, \ r_{t_{1}}$: Mean radii (mm)
${\bar{U}}_{k}, \ {\bar{W}}_{k}$: Radial and axial velocity components (mm s$^{-1}$)
$${\bar{P}}$$: Pressure (g mm$$^{-1}$$ s$$^{2}$$)
$U_{0}$: Constant secretion velocity (mm s$^{-1}$)
*K*: Permeability of the porous medium (mm$^{2}$)
